# Efficacy and safety of anti-PD-1-based therapy in combination with PARP inhibitors for patients with advanced solid tumors in a real-world setting

**DOI:** 10.1007/s00262-021-02852-4

**Published:** 2021-03-19

**Authors:** Zhaozhen Wu, Haitao Tao, Sujie Zhang, Xiao Wang, Junxun Ma, Ruixin Li, Zhefeng Liu, Jinliang Wang, Pengfei Cui, Shixue Chen, Huang Di, Ziwei Huang, Xuan Zheng, Yi Hu

**Affiliations:** 1 Department of Medical Oncology, Chinese PLA General Hospital, Beijing, 100853 China; 2 Beijing Chest Hospital, Beijing, 101149 China; 3 School of Medicine, Nankai Universitiy, Tianjin, 300071 China; 4 School of Medicine, Oregon Health & Science University, Oregon, 97239-3098 USA

**Keywords:** PARP inhibitors, Anti-PD-1, Tumors, Efficacy, Safety

## Abstract

**Background:**

Rationale exists for combining immune checkpoint inhibitors and PARP inhibitors (PARPi), and results of clinical trials in ovarian cancer are promising, but data in other cancers are limited.

**Method:**

Efficacy and safety of PARPi/anti-PD-1 in advanced solid tumors were retrospectively analyzed. The efficacy measures included objective response rate (ORR), disease control rate (DCR), progression-free survival (PFS) and overall survival (OS).

**Results:**

This retrospective study included data from 40 patients. The ORR was 27.5% (95% CI, 13.0–42.0%), with a DCR of 85.0% (95% CI, 73.4–96.6%). Except four patients in first-line treatment (three with PR and one with SD), the ORR of ≥second-line treatment, non-small cell lung cancer (NSCLC) and small cell lung cancer (SCLC) was 22.2%, 23.1% and 28.6%, and the DCR was 83.3%, 84.6% and 71.4%, separately. The median PFS of all patients, ≥second-line treatment, NSCLC and SCLC was 4.6 m, 4.2 m, 4.5 m and 3.7 m. The median OS was 9.4 m, 11.4 m, 12.7 m and 5.4 m, respectively. Multivariable analysis revealed that BRCA1/2 mutation was positively correlated with ORR (*P* = 0.008), and LDH≥250U/L was negatively correlated with lowered DCR (*P* = 0.018), while lymphocyte number, ECOG and LDH significantly influenced both PFS and OS. We found that the possible resistant mechanisms were sarcomatous degeneration and secondary mutation, including BRCA2 truncation mutation, A2M, JAK1,T790M, KEAP1 and mTOR mutation. 37.5% patients had ≥grade 3 adverse events.

**Conclusion:**

PARPi/anti-PD-1 is an effective and tolerable method for patients with advanced solid tumors, and BRCA1/2 is a potential biomarker.

**Supplementary Information:**

The online version of this article (10.1007/s00262-021-02852-4) contains supplementary material, which is available to authorized users.

## Introduction

In recent years, immune checkpoint inhibitors (ICIs) targeting PD-1/PD-L1 have achieved substantial advancements and been commonly used to treat different solid tumors. However, only a small portion of populations derive benefit, such as patients with high expression of programmed death-ligand 1 (PD-L1), high tumor mutation burden (TMB) and microsatellite instability [1]. Programmed cell death protein 1(PD-1)/PD-L1 inhibitors can revive the exhausted T cells and enhance anticancer immune response. However, according to “the Cancer-Immunity Cycle” raised by Ira Mellman [2], elimination of tumor cells by T cells is only one of these steps. To reinvigorate the response and expand the potential benefit populations of anti-PD-1/PD-L1 blockades, combined therapies should be considered. ICIs in combination with agents that target other steps may be more effective, and PARP inhibitors belong to this kind of drugs with the ability to modulate tumor microenvironment.

PARP inhibitors are agents targeting homologous recombination (HR) pathway. PARP inhibitors, such as olaparib and niraparib, function to suppress cancer development by catalytic inhibition [3], PARP trapping [4] and modulation of anticancer immune response. The rationale for PARPi in combination with PD-1/PD-L1 inhibitors mainly involves four aspects: tumor neoantigen production, increasing tumor-infiltrating lymphocytes (TILs), enhanced antigen presentation and regulation of PD-L1 and other molecules in tumor microenvironment [5]. Different preclinical studies reported that PARP inhibitors significantly increased tumor infiltrating CD4^+^/CD8^+^ T cells which were activated by the stimulator of interferon genes (STING) pathway and recruited to tumors by tumor-specific neoantigens when combined with ICIs [6, 7]. And several preclinical studies showed that PARP inhibitors combined with anti-PD-1/PD-L1 exhibited better efficacy compared with single agents, usually with up-regulation of PD-L1 via STING activation or GSK-3β inactivation [6, 8, 9], and the enhanced therapeutic efficacy was independent of BRCA status [10]. It was also reported that PARP inhibition improved the number and killing activity of natural killer cells, accompanied by increased production of TNF-α and IFN-γ [7, 11].

Up to now, there have been many clinical studies about PARPi/ICIs underway, but available results are limited. The researchers of TOPACIO trial analyzed the efficacy and safety of niraparib/pembrolizumab combination therapy for patients with recurrent ovarian cancer and triple-negative breast cancer (TNBC), the ORRs were 18% and 21%, and the DCRs were 65% and 49% [12, 13]. Olaparib/durvalumab combination therapy displayed a clinical benefit rate of 21.1%(4/19) in patients with platinum-resistant SCLC[14], and it also showed notable efficacy for metastatic castration-resistant prostate cancer with 53%(9/17) demonstrated a PSA declination more than 50% [15]. These data demonstrate promising potentials for clinical application of PARPi/ICIs, in this study, we evaluated the efficacy and safety of olaparib/niraparib combined with pembrolizumab/nivolumab in different advanced tumors and explored the potential biomarkers and possible resistant mechanisms.

## Method

### Study design

This retrospective study collected patients with advanced solid tumors who received one or more cycles of PARPi/anti-PD1-based therapy between May 2017 and July 2019. Eligible patients were screened by the electronic medical record management system from the oncology department of People’s Liberation Army General Hospital, according to the following criteria: (1) histologically confirmed metastatic solid tumors with measurable disease; (2) the response should be evaluable and survival status could be obtained by follow-up ; (3) PD-L1 expression, BRCA1/2 mutation and TMB data were available and convincing, or tissue samples could be provided to detect the above markers; (4) the PARPi/anti-PD1-based treatment therapy was discussed and decided by a multidisciplinary team (MDT). The detailed individual regimens are provided in supplementary Table 1.

To ensure data consistency, we prospectively designed our study protocol, case report form (CRF) and the standard operating procedure (SOP) of data collection. Available pre-treatment and post-two cycles treatment blood samples (*n *= 18) and tissue specimens (*n* = 25) were collected for next generation sequencing (NGS) and PD-L1 detection by immunohistochemistry (clone 22C3) after written informed consents were obtained.

### Data collection and study outcomes

Clinicopathological information and treatment data were independently sorted and extracted by two physicians and all image materials were independently assessed and analyzed by two radiologists according to Response Evaluation Criteria in Solid Tumors, version 1.1 (RECIST 1.1), and all adverse events were recorded according to the National Cancer Institute Common Terminology Criteria for Adverse Events, version 4.0. The cutoff date of data was March 18, 2020. The efficacy and outcome measures of this study included ORR (the percentage of patients with CR/PR as per RECIST 1.1), DCR (the proportion of patients with CR/PR/SD as per RECIST 1.1), PFS (time from initial treatment to disease progression/death) and OS (time from initial treatment to death). All patients were evaluable for response, and patients failing to reach PFS and OS endpoints were censored on cutoff date.

### Statistical analysis

Clinical characteristics, demographics and safety data were summarized via descriptive statistical analysis. Point estimates and two-sided 95% CIs were provided for the analysis of ORR and DCR, and Fisher’s exact test was applied to compare the difference of two groups. For survival analysis, the median value and two-sided 95% CIs were obtained by Kaplan–Meier methods with a P value determined by the log-rank test. Single factor analysis and binary logistic regression were used to sort the biomarker of response. Univariate analysis and multivariate COX regression model were used to investigate the influence factor of PFS and OS. Two-sided P values were evaluated, and *P* < 0.05 was considered statistically significant. All statistical analyses were performed using SPSS 20.0 software (IBM, SPSS, Chicago, IL, USA). TMB was calculated by maftools software (version 1.4.28). Pathway enrichment analysis was performed with g:Profiler, visualized with EnrichmentMap (Cytoscape) and interpreted using clusterMaker2 (Cytoscape).

## Results

### Patient characteristics

Between May 2017 and July 2019, 42 patients were screened, and 40 patients who met the eligibility criteria were included in this study. The median follow-up time was 289 (42–681) days, 21 patients permanently discontinued treatment because of disease progression, and 14 patients discontinued treatment due to toxicities. Of all the study populations, 13 patients were diagnosed with NSCLC, eight with SCLC, six with gynecologic tumors, four with pancreatic cancer, three with cholangiocarcinoma, two with prostate cancer, two with sarcoma, one with breast cancer and one with pleural mesothelioma. The median age was 59 years old (range, 38–81), and 26 (65.0%) patients were men. All patients had at least one metastasis, and 13 (32.5%) patients had an Eastern Cooperative Oncology Group (ECOG) performance score of 2 or 3. Among the study populations, the median treatment line was three (range 1–6), with 4 (10%) of 40 patients receiving this combination therapy as first-line treatment owing to BRCA1/2 mutation and personal condition. Baseline lymphocyte number, lactate dehydrogenase (LDH) level, PD-L1 expression, BRCA status and TMB data were collected. Twenty-five patients had available tissue specimens to confirm the PD-L1 expression and perform BRCA1/2-included NGS, and 17 patients had blood samples to evaluate the change of TMB before and after treatment. Characteristics data are summarized in Table 1.

**Table 1:** Clinical data for all patients

Characteristic	All patients (*n*=40)	≥2nd line (*n*=36)	NSCLC (*n*=13)	SCLC (*n*=7)
Median age (range),	59(31-83)	58(31-83)	57(42-76)	64(54-73)
Gender, *n* (%)				
Male	26(65.0)	24(66.7)	12(92.3)	7(100.0)
Female	14(35.0)	12(33.3)	1(7.7)	0(0.0)
ECOG, *n* (%)				
0–1	27(67.5)	23(63.9)	9(69.2)	3(42.9)
≥2	13(32.5)	13(36.1)	4(30.8)	4(57.1)
Smoking history, *n* (%)				
Current or former	17(42.5)	15(41.7)	6(46.2)	6(85.8)
Never	23(57.5)	21(58.3)	7(53.8)	1(14.2)
Metastasis number, *n* (%)				
Number < 3	28(70.0)	25(69.4)	11(84.6)	3(42.9)
Number ≥ 3	12(30.0)	11(30.6)	2(15.4)	4(57.1)
BRCA mutation status, *n* (%)				
Mutation	15(37.5)	12(33.33)	5(38.5)	1(14.2)
Wild-type	25(62.5)	24(66.67)	8(61.5)	6(85.8)
PD-L1 expression, *n* (%)				
<1%	25(62.5)	22(61.1)	7(53.8)	4(57.1)
≥1%	15(37.5)	14(38.9)	6(46.2)	3(42.9)
TMB, median (range), n (%)	6.5(0-30)	6.1(0-30)	10.8(1.1-16.1)	8.9(1.1-30)
< 10 m/Mb	26(65.0)	24(66.7)	5(38.5)	4(57.1)
≥ 10 m/Mb	14(35.0)	12(33.3)	8(61.5)	3(42.9)
Lymphocyte number, *n* (%)				
<0.8 × 10^9^/L	14(30.5)	12(33.3)	4(30.8)	2(28.6)
≥0.8 × 10^9^/L	26(65.0)	24(66.7)	9(69.2)	5(71.4)
LDH, *n* (%)				
<250U/L	28(70.0)	24(66.7)	10(76.9)	3(42.9)
≥250U/L	12(30.0)	12(33.3)	3(23.1)	4(57.1)
Treatment lines, *n* (%)				
1–2	16(40.0)	12(33.3)	2(18.2)	5(71.4)
≥3	24(60.0)	24(66.7)	11(81.8)	2(28.6)
Combined chemotherapy, *n* (%)				
Yes	22(55.0)	19(52.8)	6(46.2)	3(42.9)
No	18(45.0)	17(47.2)	7(53.8)	4(57.1)
Cycles of treatment, median (range), no.	6(1-20)	6(1-20)	6(2-20)	4(1-18)
Follow-up time, median (range), days	289(42-681)	344(42-681)	376(74-681)	162(42-550)

*NSCLC* non-small cell lung cancer;* SCLC* small cell lung cancer;* ECOG* Eastern Cooperative Oncology Group;* PD-L1* programmed death-ligand 1;* BRCA* breast cancer susceptibility gene;* TMB* tumor mutation burden;* LDH* lactate dehydrogenase

### Efficacy

**Fig. 1:**
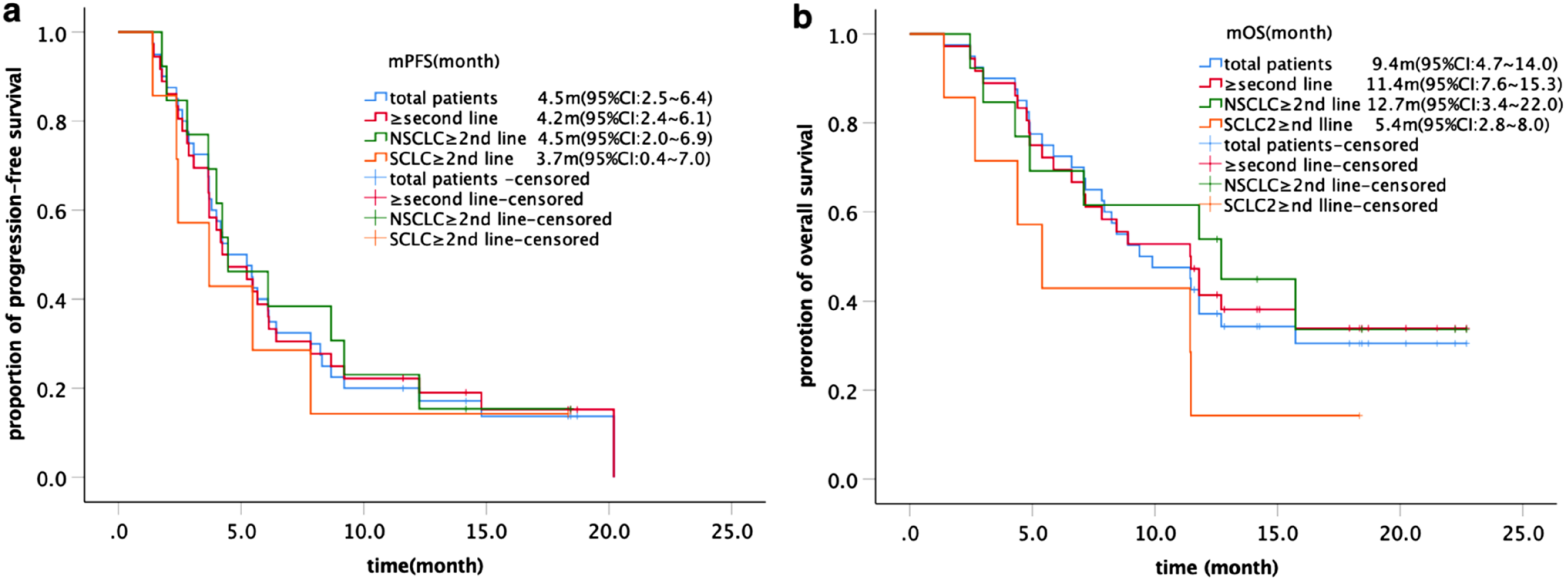
The PFS (**a**) and OS (**b**) of total populations, ≥2nd line, NSCLC and SCLC

As of March 18, 2020, all patients were evaluable, and 35 (87.5%) PFS events and 27 (67.5%) deaths had occurred. The integrate ORR was 27.5%, with a median duration of response of ≥  6.9 m (supplementary Figure 3). For four patients with BRCA1/2 mutation who received olaparib/pembrolizumab-based combined therapy as first-line treatment (the diagnosis was SCLC, rhabdomyosarcoma, cholangiocarcinoma and pancreatic cancer, respectively), disease was well controlled with three patients achieving PR and one patient achieving SD. For 36 patients in ≥second-line treatment, the ORR was 22.2% (95% CI, 8.0–36.5%), the DCR was 83.3% (95% CI, 70.5–96.1%). The ORR and DCR were 23.1% (95% CI, 5.0–53.8%) and 84.6% (95% CI, 54.6–96.1%) for NSCLC, and 28.6% (95% CI, 3.7–71.0%) and 71.4% (95% CI, 29.0–96.3%) for SCLC, separately (Table 2). The mPFS of all patients, 36 patients in ≥second-line treatment, 13 patients with NSCLC and seven with SCLC were 4.6 m (95%CI, 2.5–6.0), 4.2 m (95%CI, 2.4–6. 3), 4.5 m (95%CI, 2.0–6.9) and 3.7m (95%CI, 0.4–7.0), separately (Fig. 1a). The mOS were 9.4 m (95%CI, 4.7–14.0), 11.4m (95%CI, 7.6–15.3), 12.7 m (95%CI, 3.4–22.0) and 5.4m (95%CI, 2.8–8.0), respectively (Fig. 1b).

**Table 2:** Best overall tumor responses in the full-analysis and efficacy-evaluable populations

Populations	Total no.	CR/PR no.(%)	SD no.(%)	PD no.(%)	ORR No.(%) [95%CI]^a^	DCR No.(%)[95%CI]^b^
NSCLC	13	3(23.1)	8(61.5)	2(15.4)	23.1(5.0-53.8)	84.6(54.6-96.1)
SCLC^c^	8	3(28.6)	3(37.5)	2(28.6)	28.6(3.7-71.0)	71.4(29.0-96.3)
Gynecologic tumors^d^	6	2	4	0	33.3	100.0
Pancreatic cancer	4	1	3	0	25.0	100.0
Cholangiocarcinoma	3	1	1	1	33.3	66.7
Others^e^	6	1	5	0	16.7	83.3
First line	4	3	1	0	75.0	100.0
≥Second line	36	8(22.2)	22(61.1)	6(16.7)	22.2(8.0-36.5)	83.3(70.5-96.1)
All populations	40	11(27.5)	23(57.5)	6(15.0)	27.5(13.0-42.0)	85.0(73.4-96.6)

^a^ Include complete and partial responses

^b^ Include complete and partial responses and stable disease

^c^ Patient for first-line treatment(n=1,PR) was not included when calculated ORR and DCR

^d^ Include two cases of ovarian cancer, two of endometrial carcinoma, one of cervical carcinoma and one of fallopian tube cancer

^e^ Include two cases of prostate cancer, two of sarcoma, one of breast cancer and one of pleural mesothelioma

**Fig. 2:**
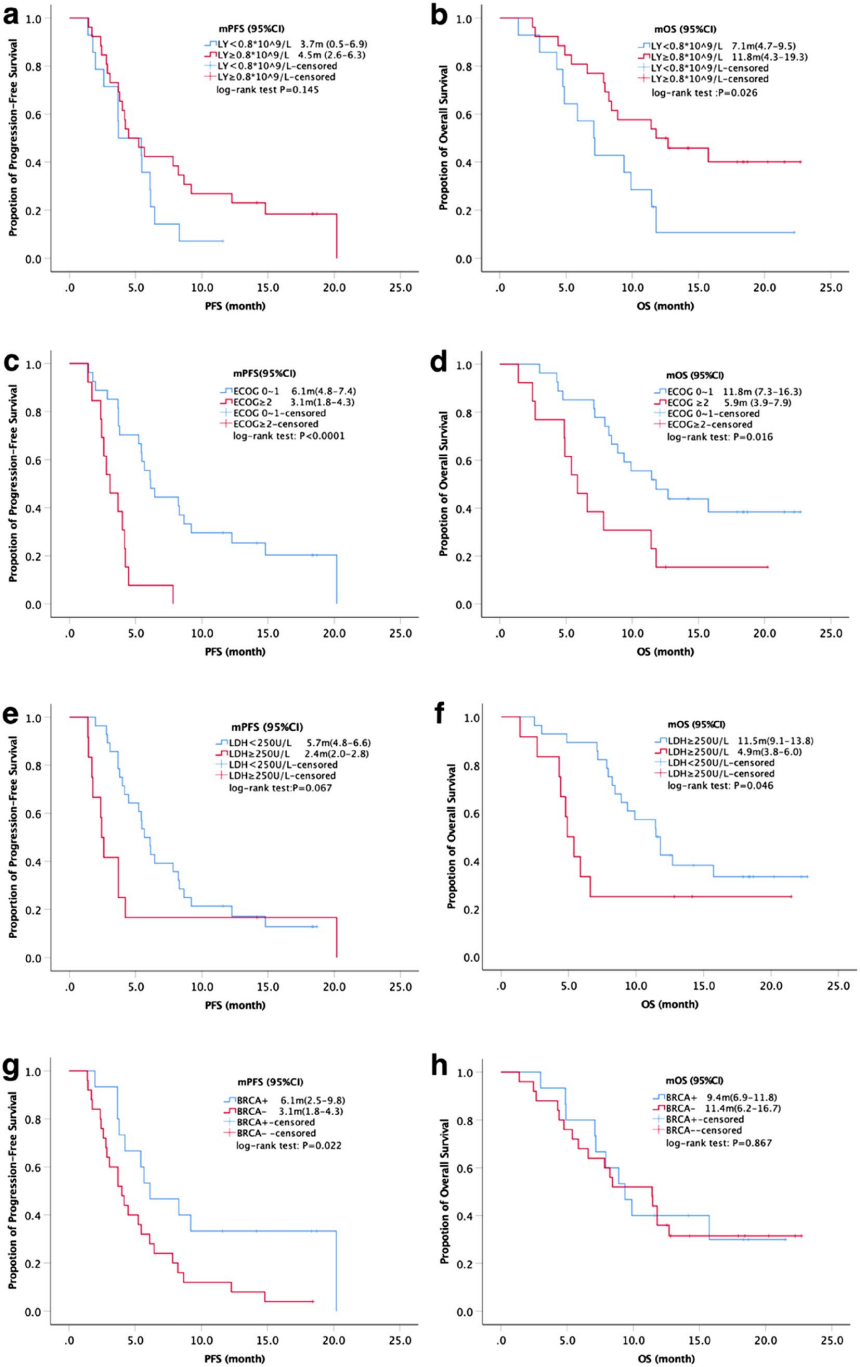
Subgroup analysis of PFS and OS. Lymphocyte number-based subgroup analysis of PFS (**a**) and OS (**b**), ECOG-based subgroup analysis of PFS (**c**) and OS (**d**), LDH-based subgroup analysis of PFS (**e**) and OS (**f**), BRCA-based subgroup analysis of PFS (**g**) and OS (**h**)

We analyzed the potential biomarkers for efficacy and prognosis, factors with *P* <0.1 for single factor analysis were included in binary logistic regression, and potential clinical biomarkers (BRCA, PD-L1 and TMB) were also included regardless of *P* values, and results of logistic regression showed BRCA1/2 mutation was positively correlated with ORR (*P* = 0.008) and LDH≥250U/L was negatively correlated with the DCR (*P* = 0.018). Results of univariate analysis and multivariate COX regression model indicated that lymphocyte number, ECOG and LDH significantly influenced both PFS and OS (*P* < 0.05, supplementary Table 2). The ORR of BRCA+ patients was 60.0% versus 8.0% (*P* = 0.001) when compared with BRCA-, while the mPFS and mOS were 6.1 m versus 4.0 m (*P* = 0.022, Fig. 2g) and 9.4 m versus 11.8m (*P*=0.867, Fig. 2h). Although PD-L1 was not shown as a predictive factor for efficacy and outcomes in our univariate and multivariate analyses, we also did PD-L1-based subgroup analysis of PFS and OS, given that PD-L1 was reported as a biomarker of PD-1 inhibitors. Results showed that the survival curves separated at about 2.5 and 5 months, respectively, then remained non-overlapping throughout the follow-up period. Compared with PD-L1<1% group, the mPFS (6.1 m vs. 4.0 m) and mOS (12.7 m vs. 8.9 m) of PD-L1≥1% group improved, still, there was no statistical difference (*P*_1_ = 0.096, *P*_2_ = 0.181, supplementary Fig. 1). Other factors-based subgroup analysis data of ORR and DCR are summarized in supplementary Table 3, and lymphocyte number, ECOG, LDH and BRCA1/2-based subgroup analyses of PFS and OS are shown in Fig. 2. Since chemotherapy could also modulate immune response, we compared the outcomes of patients between PARPi/ICI/chemotherapy group and PARPi/ICI group, and the results showed a mPFS of 4.5 m (95%CI, 2.6–6.4 m) versus 4.2 m (95%CI, 1.0–7.5 m), and a mOS of 8.9 m (95%CI, 5.2–12.6 m) versus 9.4 m (95%CI, 0.0–19.4 m) (*P*_1_ = 0.695, *P*_2_ = 0.919, supplementary Fig. 2).

**Table 3:** Treatment-related adverse events of patients with or without chemotherapy

Adverse events	Any grade no (%)			Grade ≥ 3 No (%)		
	Total	Chemo+	Chemo–	Total	Chemo+	Chemo−
Any treatment-related	38(95.0)	21(95.5)	17(94.4)	^a^ 14 (35.0)	^a^ 9(40.9)	5(27.8)
Immune-related adverse events	7(17.5)	3(13.6)	4(22.2)	3 (7.5)	1(4.5)	2(11.1)
^b^ Myelosuppression	24(60.0)	17(77.3)	7(38.9)	12(30.0)	9(40.9)	3(16.7)
Anemia	19(47.5)	13(59.1)	6(33.3)	10(25.0)	8(36.4)	2(11.1)
Leukopenia and/or neutropenia	9(22.5)	8(36.4)	1(5.6)	2(5.0)	1(4.5)	1(5.6)
Thrombocytopenia	8(20.0)	7(31.8)	1(5.6)	3(7.5)	2(9.1)	1(5.6)
Decreased appetite	8(20.0)	5(22.7)	3(16.7)	0(0.0)	0(0.0)	0(0.0)
Nausea and vomiting	8(20.0)	6(27.3)	2(11.1)	0(0.0)	0(0.0)	0(0.0)
Diarrhea	4(10.0)	1(4.5)	3(16.7)	1(2.5)	0(0.0)	0(0.0)
Abdominal pain and discomfort	3(7.5)	2(9.1)	1(5.6)	0(0.0)	0(0.0)	0(0.0)
Rash	4(10.0)	3(13.6)	1(5.6)	1(2.5)	0(0.0)	1(5.6)
Myalgia and arthralgia	3(7.5)	2(9.1)	1(5.6)	0(0.0)	0(0.0)	0(0.0)
Fever	2(5.0)	1(4.5)	1(5.6)	0(0.0)	0(0.0)	0(0.0)
Fatigue	9(22.5)	3(13.6)	6(33.3)	0(0.0)	0(0.0)	0(0.0)
Adenine increase	2(5.0)	1(4.5)	1(5.6)	0(0.0)	0(0.0)	0(0.0)
Transaminase increase	4(10.0)	2(9.1)	2(11.1)	1(2.5)	0(0.0)	1(5.6)
Hyperthyroidism	1(2.5)	0(0.0)	1(5.6)	0(0.0)	0(0.0)	0(0.0)
Hypothyroidism	2(5.0)	2(9.1)	0(0.0)	0(0.0)	0(0.0)	0(0.0)
Pneumonitis	5(12.5)	1(4.5)	4(22.2)	2(5.0)	1(4.5)	1(5.6)

^a^One death of immune-related pneumonitis was listed as grade 5 adverse events

^b^Chemotherapy may increase the total incidence of myelosuppression at all grade level (*P* = 0.023)

Analysis of patients in ≥ second-line treatment was similar (data not shown), and multivariable analysis of NSCLC and SCLC was not conducted because of small case number. Univariate analysis of NSCLC also showed that patients with BRCA1/2 mutation had a higher ORR [60% (95%CI, 31.9–88.1%) vs. 0% (95%CI, 0–36.9%), *P* = 0.035], and patients with baseline lymphocyte ≥0.8 × 10^9^/l had a better mPFS [8.7 m (95%CI, 0–20.9) vs. 2.0 m (0.1–3.8), *P* = 0.021).

### ΔTMB, molecular characteristics and possible resistance mechanisms

We compared the ΔTMB of 18 cases with response evaluation (supplementary Table S4), and we found patients with BRCA1/2 mutation had higher rate of ΔTMB>0 (66.7% vs. 11.1%, *P* = 0.206). And subgroup analysis showed ORR of patients with ΔTMB > 0 was higher than those with ΔTMB ≤ 0 (66.7% vs. 20%, *P* = 0.321), but there was no statistic difference maybe because of small case number.

**Fig. 3:**
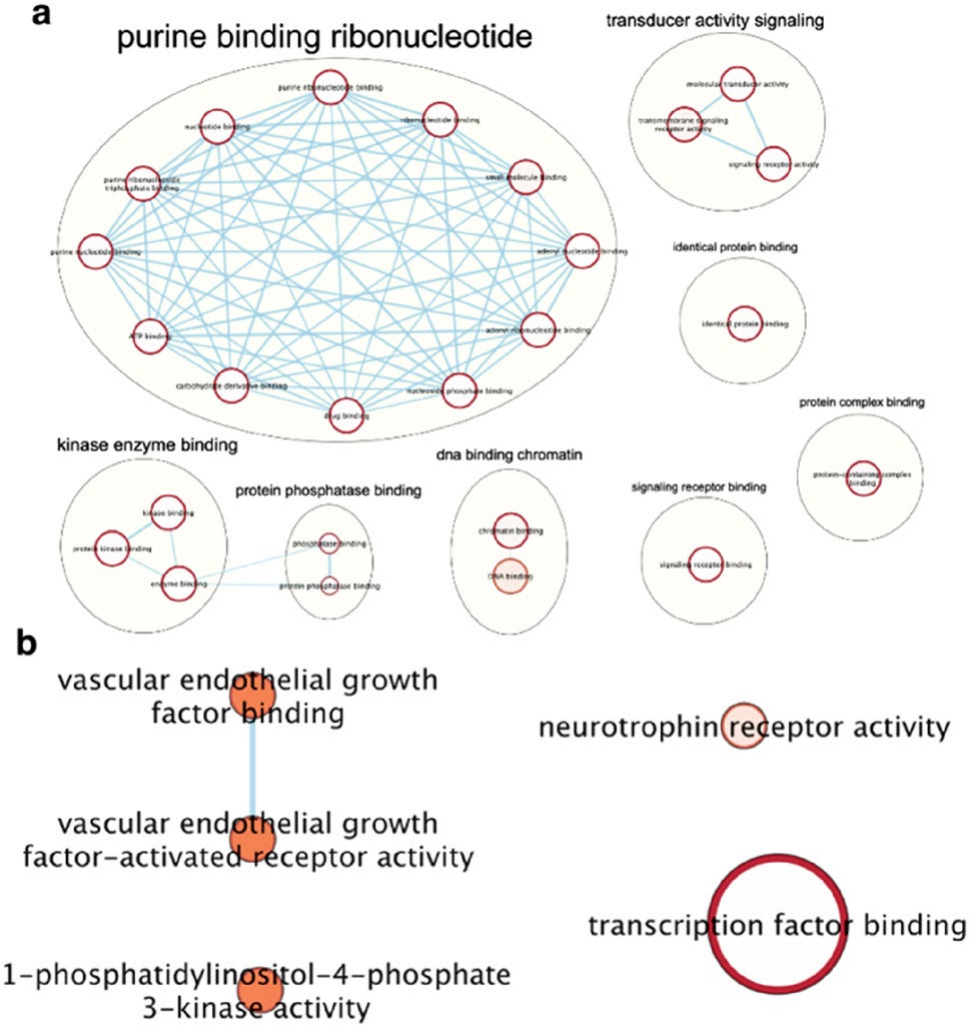
The molecular function pathways enriched in favorable-efficacy group (**a**) and unfavorable-efficacy group (**b**).** a** Favorable-efficacy group includes patients with PR/CR and SD ≥ 6 m; **b** Unfavorable-efficacy group includes patients with PD and SD < 6m

We did a comparative pathway enrichment analysis between favorable and unfavorable-efficacy groups, by Gene Ontology (GO) molecular function database. Results showed that pathways enriched in two groups were different with 12 pathways overlapped, 29 pathways only in favorable-efficacy group and five pathways only in unfavorable-efficacy group. Further interpretation found that eight clusters were only enriched in favorable-efficacy group, including “purine binding ribonucleotide,” “kinase enzyme binding,” “protein phosphatase binding,” “DNA binding,” “signaling receptor binding,” “protein complex binding,” “identical protein binding” and “transducer activity signaling” (Fig. 3a), while five pathways were only enriched in unfavorable-efficacy group, including “VEGF binding,” “VEGFR activity,” “1-phophatidylinositol-4-phosphate-3-kinase activity,” “neurotrophin receptor activity” and “transcription factor binding” (Fig. 3b).

We also summarized the molecular characteristics at disease progression and found the possible resistant mechanisms included sarcomatous degeneration and secondary gene mutation, of which one patient with pancreatic cancer was found to be with multiple BRCA2 truncation mutation, two patients with A2M mutation (SCLC and sarcoma), one with JAK1 mutation (pancreatic cancer), T790M mutation (cervical cancer), KEAP1 (prostate cancer) and mTOR mutation (NSCLC), respectively.

### Safety

The total incidence of adverse events was 95.0% (95%CI, 83.1–99.4%), and 15 (37.5%, 95%CI, 22.7–54.2%) of 40 patients had grade 3–5 treatment-related adverse events (TRAE), of whom one patient died of immune-related pneumonitis, three patients permanently discontinued treatment, and 10 patients temporarily suspended and re-challenged treatment after adverse effects were well-controlled. The overall rate of immune-related adverse events (irAEs) was 17.5%, and incidence of ≥ grade 3 irAEs was 7.5%. The most common TRAE were anemia (in 19 [47.5%] of 40 patients), fatigue (9 [22.5%]), neutropenia (9 [22.5%]), thrombopenia (8 [20%]), loss of appetite (8 [20%]), nausea and vomiting (8 [20%]). And compared with chemo- group, the incidence of myelosuppression in chemo+ group was higher (77.3% vs. 38.9%, *P* = 0.023) (Table 3).

## Discussion

Given the ability of PARP inhibitors to modify the tumor microenvironment, especially the recruitment and priming of CD4^+^ and CD8^+^T cells by producing neoantigen and releasing cytokines and chemokines, such as INF-γ, CCL5 and CXCL10 [10, 16], PD-1 inhibitors in combination with PARPi have potential to broaden durable responses and extend benefit populations of both PD-1 inhibitors and PARP inhibitors. Our study found that PARPi/anti-PD-1 combination therapy exhibited remarkable clinical efficacy in advanced solid tumors, especially BRCA1/2 mutant patients. For four patients in first-line treatment, three achieved PR and one achieved SD, and for 36 patients in ≥second-line treatment, the ORR, DCR, mPFS and mOS were 22.2%, 83.3%, 4.2 m and 11.4 m. We also found that 28.6% (2/7) of patients with SCLC (≥second line) and 33.3% (2/6) of patients with gynecologic tumors achieved PR, which was higher than single anti-PD-1 regimen in the similar settings (13.7% for SCLC [17] and<15% for gynecologic tumors [18]). The ORRs of PARPi/anti-PD-1 in both SCLC and gynecologic tumors were a little higher than other studies [12, 14], maybe owing to earlier treatment line, combined chemotherapy and different population characteristics. Since PD-1 plus chemotherapy has been well studied in NSCLC, we have tried to compare the response rates of this combination with PARPi in addition to PD-1/chemotherapy, but different treatment lines lead to a limitation. Binary logistic regression analysis indicated that BRCA status was a biomarker for response, and subgroup analysis showed better ORR in BRCA+ group for both total efficacy-evaluable populations and NSCLC, which was in accordance with other study [13].

Survival analysis in our study showed longer mPFS but shorter mOS for BRCA+ when compared with BRCA−, however, statistic difference was seen only in mPFS (6.1 m vs. 3.1m, *P* = 0.022) but not mOS (9.4 m vs. 11.4 m, *P* = 0.867), which might due to small case number and different cancer types between BRCA+ and BRCA^−^ group. The subgroup analysis of PARPi/ICIs combination in other studies showed longer mPFS for BRCA+ in TNBC cohort instead of ovarian cancer [12, 13], and no data of mOS were reported, therefore, it will be hard to sort out if the difference is due to the biomarker or cancer types, and prospective studies with larger number of patients and specific cancer type are needed. Despite that results of binary logistic regression showed TMB had significant effect on ORR (*P* = 0.045), subgroup analysis by TMB showed no statistic difference (19.2% vs. 42.8%, *P* = 0.148), and there was no statistic difference of ORR between patients with ΔTMB > 0 and ΔTMB ≤ 0. We thought that small case number was a reason, and the time to collect blood samples should be adjusted. We also did PD-L1-based subgroup analysis of PFS and OS, although the survival curves separated and the mPFS and mOS of PD-L1≥1% group improved, still statistical difference was not found. Chemotherapy used to be considered to promote the response of ICIs to cancer [19], but in our study, chemotherapy-based subgroup analysis of PFS and OS showed no statistical difference, and this was in accordance with two recent studies, which indicated that chemotherapy weakly contributed to predicted neoantigen expression in ovarian cancer [20], and that chemotherapy exerted unfavorable influence on subsequent immunotherapy by inducing a decrease in tumor mutation burden [21]. Therefore, the effect of chemotherapy on ICIs should be further studied. In addition, maybe PARP inhibitors synergized with PD-1 blockade mainly by regulating the immune context, especially TILs, and in our study, baseline lymphocyte was found to markedly influence both PFS and OS, therefore, further dynamic detection of lymphocyte immunophenotyping is important.

We did molecular function pathway enrichment analysis with GO database, and VEGF/VEGFR pathways were found enriched in unfavorable-efficacy group, and this provided an insight for us to appropriately combine PARPi/anti-PD-1 with anti-angiogenesis drugs according to patients’ clinical condition. Meanwhile, favorable-efficacy group was more enriched in purine/DNA/kinase/transducer signaling receptor binding associated pathways, and it was in accordance with “BRCA mutation was a biomarker for response,” therefore, it is worthy to explore if it is feasible to add platinum to this combined therapy in BRCA1/2 wild-type tumors to increase DNA damage pressure. Besides, some of the patients conducted NGS at the time of PD, and we identified distinct gene mutation compared with pre-treatment, such as secondary BRCA1/2 truncation mutation, A2M, JAK1, KEAP1, T790M and mTOR, of which BRCA2 truncation mutation was reported to be an important resistance mechanism of PARP inhibitors [22], and JAK1 truncating mutation was recognized as an acquired resistance mechanism of PD-1 inhibitors owing to a lack of response to interferon gamma [23]. A2M mutation was related to inflammatory cascades and might facilitate cancer development by decreasing the expression of CD29 and CD44 [24], in addition, T790M, mTOR mutation and KEAP1 loss were identified as resistance mechanisms [25]. However, our small study only verified that multiple secondary BRCA2 mutation was a resistance mechanism of PARPi/PD-1 blockade therapy [26], other putative mechanisms should be further verified.

In the perspective of safety, the total adverse events were similar with other studies [12, 13], and the irAEs did not increase compared with ICIs [27], but the incidence of myelosuppression was a little higher, which was probably due to the additional utilization of chemotherapy. Based on our data and other clinical investigations, we suggest that PARPi/ICIs-related clinical trial is a consideration for advanced tumors without standard treatment, especially for those selected patient population, such as BRCA1/2 mutation. Still, there were some limitations in our study, for example, we did not distinguish germline or somatic mutation, because there were only two cases with germline BRCA1/2 mutation, so BRCA mutation forms and other DNA damage-related mutations, particularly HR deficiency, should also be considered. Meanwhile, combination treatment drugs in our study were not entirely the same, and part of PD-L1 expression and NGS data was collected from other platforms. In addition, comparison with PD-1 inhibitors alone should be further done. Therefore, prospective clinical studies are needed to better understand the anti-tumor effect of PARPi/anti-PD-1 combination, and further analysis of biomarker and immunophenotyping is important.

## Supplementary Information

Below is the link to the electronic supplementary material.

Supplementary file 1 (PDF 401kb)

## Data Availability

Not applicable.

## Abbreviations

BRCA: Breast cancer susceptibility gene
CXCL10: C–X–C motif chemokine ligand 10
CCL5: C–C motif chemokine ligand 5
DCR: Disease control rate
ECOG: Eastern Cooperative Oncology Group
ICIs: Immune checkpoint inhibitors
HR: Homologous recombination
LDH: Lactate dehydrogenase
NSCLC: Non-small cell lung cancer
ORR: Objective response rate
OS: Overall survival
PARP: Poly (ADP-ribose) polymerase
PFS: Progression-free survival
PD-1: Programmed cell death protein 1
PD-L1: Programmed death-ligand 1
SCLC: Small cell lung cancer
STING: The stimulator of interferon genes
TNBC: Triple-negative breast cancer
TILs: Tumor-infiltrating lymphocytes
TMB: Tumor mutation burden
