# Supplementary material for: Efficacy and safety of anti-PD-1-based therapy in combination with PARP inhibitors for patients with advanced solid tumors in a real-world setting
Source: Cancer Immunol Immunother. 2021 Mar 19;70(10):2971–80. doi: 10.1007/s00262-021-02852-4 (PMC8423634; doi:10.1007/s00262-021-02852-4)
Supplement: Supplementary file 1 — (PDF 401kb) [file 262_2021_2852_MOESM1_ESM.pdf]

No.	Cancer type	Treatment line	PARP inhibitors	PD-1 inhibitors	combined therapy	cycles
1	Pancreatic cancer	2	olaparib 400mg bid	pembrolizumab 200mg/3w	cisplatin120mg/3w	5
2	NSCLC	5	olaparib 400mg bid	nivolumab 240mg/2w		6
3	NSCLC	4	olaparib 400mg bid	nivolumab 240mg/2w		2
4	SCLC	2	olaparib 400mg bid	nivolumab 240mg/2w		3
5	SCLC	1	olaparib 300mg bid	pembrolizumab 200mg/3w	etoposide 200/200/100mg day1-3 + cisplatin120mg/3w* 6 cycles	10
6	Endometrial carcinoma	4	olaparib 300mg bid	pembrolizumab 200mg/3w	cisplatin120mg/3w	4
7	Cervical carcinoma	2	olaparib 300mg bid	nivolumab 140mg/2w		4
8	Cholangiocarcinoma	3	olaparib 300mg bid	pembrolizumab 200mg/3w		2
9	SCLC	5	olaparib 300mg bid	pembrolizumab 200mg/3w		2
10	NSCLC	3	olaparib 400mg bid	nivolumab 240mg/2w		4
11	SCLC	2	olaparib 300mg bid	pembrolizumab 200mg/3w		10
12	SCLC	2	olaparib 300mg bid	pembrolizumab 200mg/3w	cisplatin 100mg/3w	1
13	Prostate cancer	4	olaparib 300mg bid	pembrolizumab 200mg/3w	albumin-binding paclitaxel 100mg/3w	3
14	SCLC	3	olaparib 300mg bid	pembrolizumab200mg/3w	cisplatin120mg/3w	6
15	Sarcoma	1	olaparib 300mg bid	pembrolizumab200mg/3w		6
16	NSCLC	6	olaparib 300mg bid	pembrolizumab 200mg/3w		2
17	Cholangiocarcinoma	1	olaparib 300mg bid	pembrolizumab 100mg/3w	oxaliplatin150mg/3w*6 cycles	10
18	NSCLC	3	olaparib 300mg bid	pembrolizumab 200mg/3w	cisplatin120mg/3w*6 cycles	10
19	Pancreatic cancer	1	olaparib 300mg bid	pembrolizumab 200mg/3w	cisplatin110mg/3w	4
20	NSCLC	5	olaparib 300mg bid	pembrolizumab 200mg/3w	cisplatin 120mg /3w	4
21	Prostate cancer	3	olaparib 300mg bid	nivolumab 240mg/2w		8
22	Cholangiocarcinoma	3	olaparib 300mg bid	pembrolizumab 200mg/3w	oxaliplatin150mg/3w	6
23	NSCLC	3	niraparib 300mg qd	pembrolizumab 200mg/3w	cisplatin 100mg/3w	5

24	SCLC	2	olaparib 300mg bid	pembrolizumab 200mg/3w	cisplatin110mg/3w	4
25	NSCLC	3	olaparib 300mg bid	pembrolizumab 200mg/3w		16
26	Fallopian tube cancer	5	niraparib 300mg qd	pembrolizumab 200mg/3w	cisplatin 100mg/3w	6
27	Pancreatic cancer	3	olaparib 300mg bid	pembrolizumab 200mg/3w	oxaliplatin250mg/3w	4
28	Sarcoma	4	olaparib 300mg bid	pembrolizumab 200mg/3w	albumin-binding paclitaxel 200mg/3w	2
29	NSCLC	4	olaparib 300mg bid	pembrolizumab 200mg/3w		10
30	NSCLC	2	olaparib 300mg bid	pembrolizumab 200mg/3w	cisplatin 50mg/3w	6
31	Pleural mesothelioma	3	niraparib 300mg qd	pembrolizumab 200mg/3w		6
32	NSCLC	5	olaparib 300mg bid	pembrolizumab 200mg/3w	cisplatin 70mg/3w	6
33	Endometrial carcinoma	6	olaparib 300mg bid	nivolumab 240mg/2w		4
34	Pancreatic cancer	2	olaparib 300mg bid	pembrolizumab 200mg/3w		18
35	Breast cancer	2	olaparib 300mg bid	pembrolizumab 200mg/3w	bevacizumab500mg/3w	20
36	NSCLC	3	olaparib 300mg bid	nivolumab 200mg/3w	cisplatin 100mg/3w*6 cycles	14
37	Ovarian cancer	4	niraparib 300mg qd	pembrolizumab 200mg/3w	cisplatin 60mg/3w*6 cycles	14
38	NSCLC	2	olaparib 300mg bid	pembrolizumab 200mg/3w		20
39	Ovarian cancer	2	olaparib 300mg bid	pembrolizumab 200mg/3w	bevacizumab 300mg/3w	20
40	SCLC	2	olaparib 300mg bid	pembrolizumab 200mg/3w		18

Supplementary Table 1 The detailed regimens of 40 patients receiving PARPi/anti-PD-1 therapy.

NSCLC, non-small cell lung cancer; SCLC, small cell lung cancer.

Clinical factors	P values for ORR		P values for DCR		P values for PFS		P values for OS	
	Univariable	Multivariable	Univariable	Multivariable	Univariable	Multivariable	Univariable	Multivariable
gender	0.396		0.926		0.764		0.494	
Age	0.138		0.526		0.845		0.677	
Smoking history	0.629		0.624		0.924		0.754	
ECOG	0.08	0.328	0.33		0.000	**0.001**	0.02	**0.028**
Metastasis number	0.105		0.05	0.146	0.001	0.086	0.068	0.694
BRCA mutation status	0.002	**0.008**	0.276	0.962	0.026	**0.059**	0.867	0.528
PD-L1 expression	0.135	0.074	0.819	0.967	0.102	0.952	0.188	0.896
TMB	0.118	**0.045**	0.41	0.325	0.416	0.702	0.734	0.876
Treatment line	0.253		0.232		0.074	0.258	0.666	
Combined therapy	0.174		0.261		0.696		0.919	
Lymphocyte number	0.53		0.41		0.151	**0.041**	0.031	**0.003**
LDH	0.105		0.012	**0.018**	0.072	**0.041**	0.052	**0.026**

Supplementary Table 2 Analysis of factors affecting the efficacy and outcomes of PARPi/anti-PD-1 therapy

ORR, objective response rate; DCR, disease control rate; PFS, progression-free survival; OS, overall survival; ECOG, Eastern Cooperative Oncology Group; BRCA, breast cancer susceptibility gene; PD-L1, programmed death-ligand 1;TMB, tumor mutation burden; LDH, Lactate dehydrogenase.

Biomarker	ORR No.(%)[95%CI]	P values	Biomarker	DCR No.(%)[95%CI]	P values
BRCA+	60.0（32.3-83.7）	**0.002**	BRCA+	93.3（68.1-99.8）	0.381
BRCA-	8.0（1.0-26.0）		BRCA-	80.0（59.3-93.2）	
TMB<10m/Mb	19.2（6.6-39.4）	0.147	LDH<250U/L	96.4（81.7-99.9）	**0.006**
TMB≥10m/Mb	42.9（17.7-71.1）		LDH≥250U/L	58.3（27.7-84.8）	

Supplementary Table 3 Biomarker-based efficacy analysis of PARPi/anti-PD-1 therapy.
ORR, objective response rate; DCR, disease control rate; BRCA, breast cancer susceptibility gene; LDH, Lactate dehydrogenase.

Patient number	TMB_1	TMB_2	ΔTMB	efficacy
1	0.00	1.08	1.08	PD
2	6.45	8.608	2.15	PD
3	2.15	2.15	0.00	SD
4	1.08	9.68	8.60	SD
5	8.60	7.52	-1.08	PD
6	6.45	6.45	0.00	SD
7	19.35	20.43	1.08	PR
8	0.00	2.15	2.15	PR
9	7.53	0.00	-7.53	SD
10	8.60	7.52	-1.08	SD
11	0.00	2.15	2.15	PR
12	1.08	3.23	2.15	PD
13	10.75	10.75	0.00	SD
14	7.53	0.00	-7.53	SD
15	4.30	9.68	5.38	PR
16	16.13	15.05	-1.08	PR
17	3.23	1.08	-2.15	PR
18	2.15	2.15	0	SD

Supplementary Table 4 ΔTMB of before and after-treatment in blood samples-available population
TMB, tumor mutation burden; $\Delta TMB = TMB_2 - TMB_1$.

Characteristic	PARPi/ICI/chemotherapy (n=22)	PARPi/ICI (n=18)	P value
Age, n (%)			0.173
<65	18(81.8)	11(61.1)	
≥65	4(28.2)	7(38.9)	
Gender, n (%)			
Male	13(59.1)	13(72.2)	0.510
Female	9(40.9)	5(21.8)	
ECOG, n (%)			
0–1	17(77.3)	10(55.6)	0.185
≥2	5(22.7)	8(44.4)	
Smoking history, n (%)			
Current or former	14(63.6)	9(50.0)	0.523
Never	8(36.4)	9(50.0)	
Metastasis number, n (%)			
Number<3	16(72.7)	12(66.7)	0.738
Number≥3	6(27.3)	6(33.3)	
BRCA status, n (%)			
Mutation	10(45.5)	5(27.8)	0.332
Wild-type	12(54.5)	13(72.2)	
PD-L1 expression, n (%)			
< 1%	15(68.2)	10(55.6)	0.517
≥1%	7(31.8)	8(44.4)	
TMB, n (%)			
< 10m/Mb	15(68.2)	11(61.1)	0.744
≥10m/Mb	7(31.8)	7(38.9)	
Lymphocyte number, n (%)			
< $0.8*10^9$/L	9(40.9)	5(27.8)	0.510
≥$0.8*10^9$/L	13(59.1)	13(72.2)	
LDH, n (%)			
< 250U/L	15(68.2)	13(72.2)	1.000
≥250U/L	7(31.8)	5(27.8)	

Supplementary Table 5 Clinical characteristics of chemotherapy-based subgroups

ECOG, Eastern Cooperative Oncology Group; BRCA, breast cancer susceptibility gene; PD-L1, programmed death-ligand 1; TMB, tumor mutation burden; LDH, Lactate dehydrogenase.

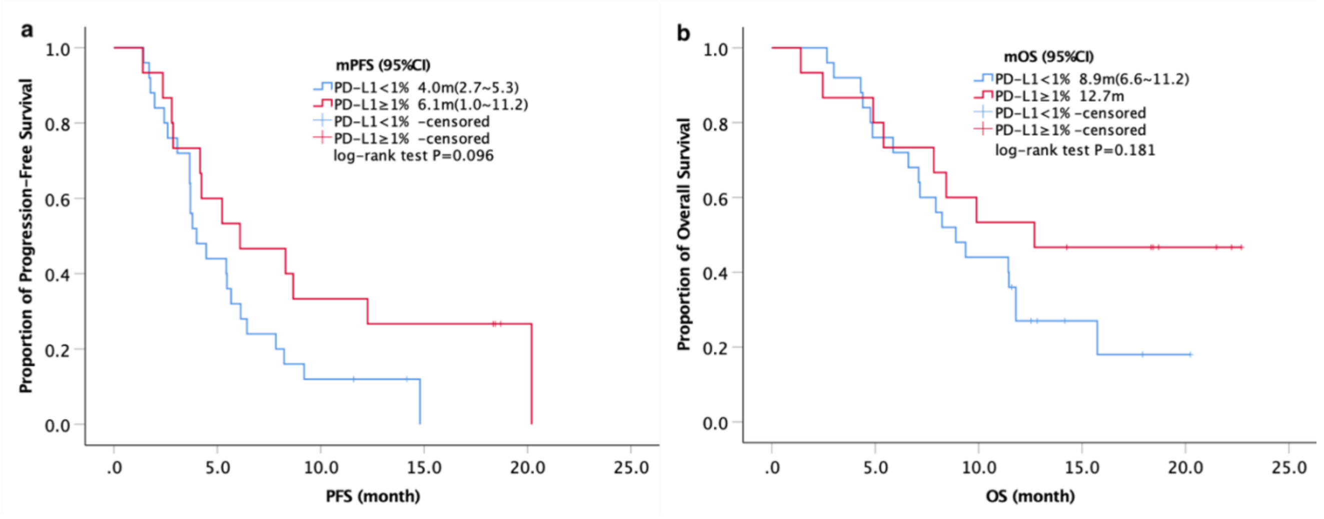

Supplementary Figure 1 PD-L1-based subgroup analysis of PFS (a) and OS (b)

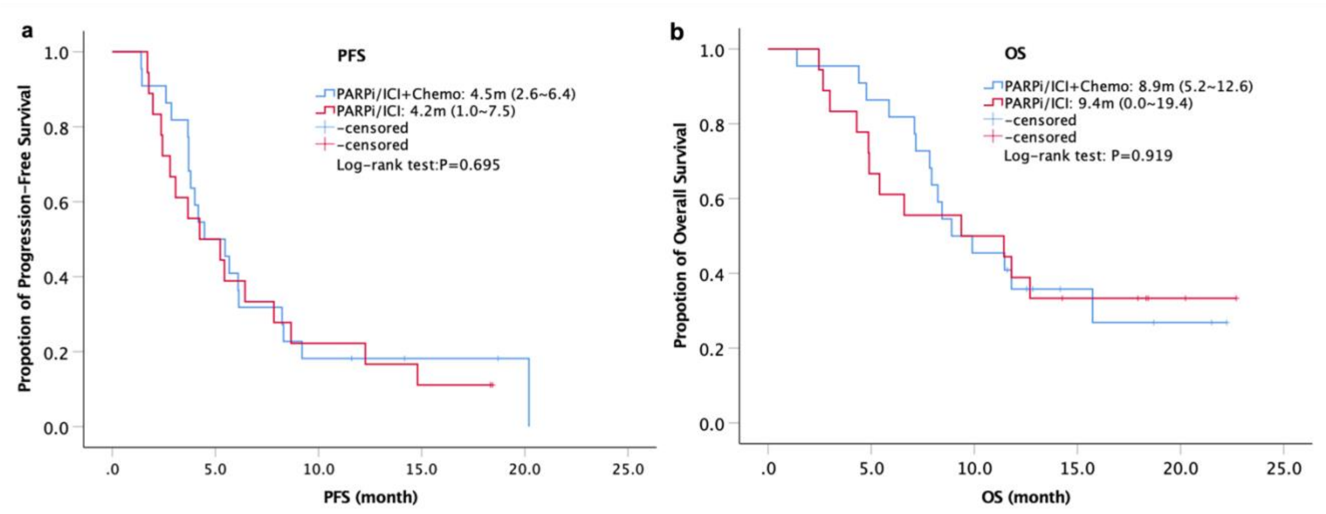

Supplementary Figure 2 Chemotherapy-based subgroup analysis of PFS **(a)** and OS **(b)**

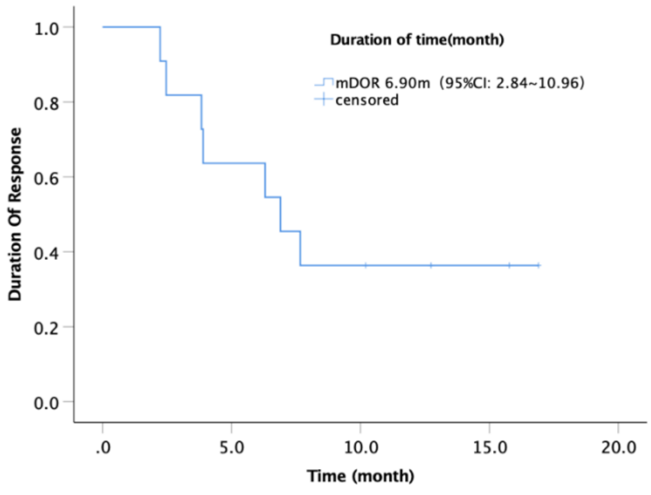

Supplementary Figure 3 Duration of response in efficacy-evaluable population.
